# How much does pre-trial testing influence complex intervention trials and would more testing make any difference? An email survey

**DOI:** 10.1186/1471-2288-6-28

**Published:** 2006-06-22

**Authors:** Shaun Treweek, Frank Sullivan

**Affiliations:** 1 Tayside Centre for General Practice, Community Health Sciences Division, University of Dundee, Kirsty Semple Way, Dundee DD2 4BF, UK

## Abstract

**Background:**

The UK Medical Research Council has proposed that complex interventions should be tested in exploratory trials prior to a full-scale trial so as to better define the intervention and test the feasibility of components such as recruitment. It is not clear to what extent this is being done. This study aimed to determine to what extent complex interventions are tested prior to a full-scale trial and whether more or different testing would have led to a different intervention being used in the trial.

**Methods:**

Email survey of the authors of complex intervention trials published in seven major journals in 2004.

**Results:**

72% (50/69) of eligible authors replied. Eight authors did not consider their interventions to be complex. The majority of respondents' complex interventions were tested (34/42): some extensively. Conversely, only 17 of the 34 published reports describing these trials mention testing. Two-thirds (22/34) of those testing their interventions did not believe that more or different testing would have produced a more effective intervention. 31% (13/42) of all authors did believe further testing would have led to improvements. Five respondents mentioned a lack of funding as a reason for not doing more testing.

**Conclusion:**

Complex interventions are generally tested prior to their evaluation in a full-scale trial, although the amount of testing varies. Testing is often not described in trial reports, which makes it hard to judge whether a trial result could be improved with a better intervention, or whether further work with a different intervention is required.

## Background

In 2000 the UK Medical Research Council published a structure for evaluating complex interventions, which proposed that interventions should be modelled and then tested prior to a full-scale trial [1]. The pre-trial work could be expected to provide a more realistic estimate of the likely effect of the intervention and inform decisions regarding intervention design and delivery. In principle, such work may help to avoid wasting resources evaluating ineffective interventions because rigorous development and testing will reject poor interventions and unfeasible trials before they reach full-scale evaluation [2,3].

The aim of the current study was twofold. Firstly, we wanted to survey the estimates of treatment effect and recruitment given in trial reports and compare them with those actually achieved, together with any mention of piloting or testing. Secondly, we wanted to ask the authors of these trial reports whether testing did influence the design of their trials and whether more or different testing would have led to a different intervention being developed. This paper deals with the second of these; the first is covered in a sister paper [4].

## Methods

We searched the 2004 issues of the British Journal of General Practice (BJGP), the BMJ, Family Practice, the Journal of the American Medical Association (JAMA), the Journal of the American Medical Informatics Association (JAMIA), the Journal of Health Services Research and Policy (JHSRP) and the Lancet for articles indexed in Pubmed as 'randomized controlled trials'. We did this by using Pubmed's 'Limits' facility to restrict the type of article retrieved to randomized controlled trials. These journals were selected because they are high impact journals with a history (in our experience) of publishing reports of randomised controlled trials. We limited ourselves to these seven journals because we thought it would be unwise to commit resources to a systematic review without having a much better idea of the type and extent of pre-trial testing done in complex intervention studies. The current study can be seen as a pilot that can inform the development of a search strategy, inclusion criteria and selection of outcome measures for a larger systematic study.

We had two further inclusion criteria:

• the intervention must be complex

• trials must address patient care or provision of care by health professionals

To select complex intervention trials we used the MRC definition of a complex trial: '*Complex interventions in health care, whether therapeutic or preventative, comprise a number of separate elements which seem essential to the proper functioning of the intervention although the active ingredient of the intervention that is effective is difficult to specify*. ' [1].

This definition is open to interpretation. Interventions that we considered complex under this definition included the use of volunteer counsellors to increase breast-feeding, a behavioural intervention to reduce acquisition of HIV among homosexual men and the use of a structured shared care model involving education, a nurse specialist, locally agreed treatment protocols and improved primary care-secondary care communication to improve the management of diabetes in primary care. Interventions that we did not consider complex included a comparison of five antimicrobial regimens for mild to moderate facial acne, a study of a computerised guideline system that measured physician knowledge but not the care provided, using an invitation to ultrasound screening to reduce mortality from abdominal aortic aneurysm and adenotonsillectomy compared with watchful waiting in the treatment of mild symptoms of throat infections or adenotonsillar hypertrophy. These and similar interventions were excluded because they involved a single component where the active ingredient was clear or because they did not involve patient care.

Abstracts were scanned for relevance by both authors and relevant abstracts were discussed and any disagreements resolved. The full text of included studies was obtained and ST did data extraction. Of the 318 articles identified by our search, 70 met our inclusion criteria (28 from the BMJ, 17 from JAMA, ten from the Lancet, eight from Family Practice, seven from BJGP and none from both JAMIA and JHSRP. A five-question email questionnaire was sent to the corresponding author of each of the 70 included studies. The questionnaire was piloted on colleagues prior to its use in the survey although no changes to the questionnaire's wording or general design were suggested. A full copy of the questionnaire is given in the Appendix. The email sent to authors also assured them that they would remain anonymous in any future publications. A single reminder was sent one week after the initial email.

## Results

One email was incorrect and an alternative address could not be found; 72% (50/69) of the remaining authors replied but eight of these did not consider their interventions to be complex. The results presented below come from the responses of the remaining 42 authors.

The majority of interventions were tested (34/42 or 81%; 95% confidence interval = 67% to 90%), some extensively (Table 1). Conversely, only 17 (or 50%; 95% Cl = 34% to 66%) of the 34 trials that involved testing actually mention this testing in the published report. Those not mentioning their testing included some studies that ran substantial testing programs prior to the intervention's evaluation in a full-scale trial. Of the 34 respondents who said their interventions were tested, 22 (or 65%; 95% Cl = 48% to 79%) did not believe that different or further testing would have produced a more effective intervention. Four of those who did no testing thought that more testing would have improved their intervention. The 13 respondents who did believe further testing would have led to improvements gave the following reasons: better intervention (five respondents); better delivery (two); better intervention and delivery (two); more understanding of how intervention components work (one); and unspecified (three). Five respondents mentioned a lack of funding as a reason for not doing more testing. Table 2 presents some illustrative comments from these 13 respondents; almost all those saying that different or further testing would not have produced a more effective intervention simply replied 'No'.

**Table 1:** The degree and influence of pre-trial testing for 42 complex intervention trials.

	**Would more or better testing have produced a more effective intervention?**			
**Was the intervention tested?**^1^	**Yes**	**No**	**Unclear**	**Totals**
Yes – extensively	4	11	1	16
Yes – to some degree	5	11	2	18
No	4	4	0	8

**Totals**	13	26	3	42

	**Was the test phase mentioned in the published article?^2^**			
**Was the intervention tested?**^1^	**Yes**	**No**		Totals

Yes – extensively	10	6		16
Yes – to some degree	7	11		18
No	-	8		8

**Totals**	17	25		42

^1 ^Testing was categorised (by ST) as 'extensive' if the respondent considered the study a pilot, or mentioned substantial experience with similar interventions, tests of the complete intervention package running for several months, previously published pilot work, exploratory trials or the respondent described testing as extensive. All other testing was categorised as 'to some degree'.

^2 ^Done by checking the text of the respondent's 2004 publication identified by our search.

**Table 2:** Would more testing have made a difference?

"Yes, absolutely. Legal constraints prevented proper implementation of the supply-side intervention. This constraint should have been detected (and resolved) before the [start of the trial]"
"...the number of young children eligible for the program and tracked by the information system degraded over time. Proper piloting of both the information system and the public communication campaign should have reduced this attrition."
"Piloting of recruitment and assessment and compliance rates would have been helpful but no funding for time to do this."
"I think there are ways that we could have improved the assistance package, but it was ready to be tested at the time we did the intervention trial."
"A full pilot phase would have alerted us to some of problems encountered such as inadequate team working."
"Almost certainly. The more the better really – in particular in depth interviews with providers and recipients of the pilot intervention or piloting in more than one setting could have resulted ultimately in an even more robust intervention."
"With hindsight what might have been useful would have been more data on exactly what happened in the black boxes to see which components were responsible for any benefits gained."
"Answer has to be yes but only if one had enough funds and time to do multiple tests."

The testing that was done influenced the interventions in a variety of ways. Some of the testing would be relevant for any intervention trial (eg. testing of data collection instruments or training manuals) and not just those involving a complex intervention. Reported testing could be categorised as: iterative refinements of the intervention and/or its delivery (20 respondents; altered the trial design (three); altered both intervention and trial design (three); confirmed the feasibility of the trial (three); changed nothing (two); and unclear what influence testing had (three). Where trial design was changed, pre-trial testing led to an arm of the trial being dropped in three cases. Examples of how testing influenced the full-scale trial are given in Table 3. The type of information we received from respondents is very close to the short statement format given in Table 3.

**Table 3:** Examples of intervention testing and how this testing influenced the final intervention and trial.

Ways in which the intervention was tested	Ways in which testing influenced the intervention or its delivery
A component of the intervention, the guideline flowchart, was piloted in a single hospital.	The sequence and flow of questions and recommendations was altered. Some minor changes to the wording and format of the guidelines were made.
Individual components of the study (eg. data collection, intervention, retention strategies) were tested, followed by a small feasibility trial of the whole intervention package with, finally, a pilot study involving the target population.	The data collection instruments and the intervention were modified. Recruitment and retention protocols were also modified.
The computer-based decision support system was user-tested prior to the trial.	Improvements to navigation and the user interface were made.
A one-year before-after pilot of the full intervention.	Confirmed that the intervention was promising. Highlighted communication problems between different health professionals, which were addressed before the trial. An extra member of staff (a care coordinator) was added to the support team that formed part of the intervention.
Three educational videos were shown to people to get comments on their potential as an educational intervention.	Using videos as an intervention was abandoned and a completely new intervention was designed.
Educational outreach and the reminder system were piloted in one geographical area.	Feasibility of this form of intervention was confirmed.
A one-year randomised controlled feasibility study of the full intervention.	One comparison arm of the trial was dropped. Training manuals were modified slightly.

## Discussion

Most of the complex interventions evaluated by the 42 respondents were tested to some extent but this work does not always find its way into published trial reports, which leads to an incomplete description of the rationale for intervention choice. There were no clear differences between the trials of responders and non-responders although only five of the 19 non-responders (or 26%) mentioned testing in the trial report, which is about two-thirds the rate of responders (40%). It is possible that complex interventions are tested somewhat less than our results suggest because of responder bias although we have no way of knowing this. However, we believe that such a bias, if it exists, will reduce but not reverse our main finding: that most complex interventions are tested but that this testing is often not reported.

For responders, even extensive development work involving randomised controlled trials or multi-site pilots running for many weeks can fail to be mentioned in the trial report. This can be due to space restrictions imposed by journals (several respondents cited this problem), or a belief that early development work is unlikely to be considered interesting enough for journals to publish. This is a shame since without this information it may not be possible to judge whether a trial result could be improved with a better intervention, whether the intervention and its delivery are already optimal, or whether further work with a different intervention is required.

When there is testing, it is no surprise that this generally leads to changes to the intervention and/or its delivery. Testing can also lead to substantial changes to trial design. A minority of trialists neither tested their interventions nor believed that more testing would have been beneficial. Most trialists do some testing and even modest testing lasting a few weeks can refine an intervention and its delivery. If funders were more receptive to supporting, or even insisted upon, comprehensive pre-trial testing it is reasonable to believe that fewer suboptimal complex interventions would enter full-scale trials. The importance of publishing this pre-trial development work should also be acknowledged and supported by journal editors, perhaps in the electronic versions of their journals [5].

## Conclusion

Complex interventions are generally tested prior to their evaluation in a full-scale trial, although the amount of testing varies. Testing is often not described in trial reports, which makes it hard to judge whether a trial result could be improved with a better intervention, or whether further work with a different intervention is required.

## Competing interests

The author(s) declare that they have no competing interests.

## Authors' contributions

ST had the initial idea for the study, designed the survey instrument, did the survey and wrote the initial draft of the paper. FS contributed to the development of the survey instrument and contributed to and edited the paper. ST acts as guarantor for the paper.

## Appendix

The questionnaire used in the email survey is given below.

Study title: [Title]

Question 1: Was your intervention complex?

The Medical Research Council uses the following definition of a complex intervention [Medical Research Council. A framework for development and evaluation of RCTs for complex interventions to improve health. April 2000.]:

'Complex interventions in health care, whether therapeutic or preventative, comprise a number of separate elements which seem essential to the proper functioning of the intervention although the active ingredient of the intervention that is effective is difficult to specify.'

With this in mind, do you think that your study involved a complex intervention? [Response]

Question 2: Testing or piloting the intervention

Can you briefly describe how the intervention was tested or piloted prior to being evaluated in your trial? We are particularly interested in knowing whether the full intervention was tested, or whether individual components were tested in isolation. Pilot studies investigating practical issues related to the trial (eg. recruitment rate, data collection systems, compliance) are also of interest.

[Response]

Question 3: Using the results of the test or pilot

How did the testing or pilot work influence the intervention used in the final trial?

[Response]

Question 4: Would more testing have made a difference?

Do you think that more (or different) testing would have resulted in a more effective intervention than the one used in your trial?

[Response]

Question 5: Any other comments

[Response]

## Pre-publication history

The pre-publication history for this paper can be accessed here:


